# The Human and Mouse Islet Peptidome: Effects of Obesity and Type 2 Diabetes, and Assessment of Intraislet Production of Glucagon-like Peptide-1

**DOI:** 10.1021/acs.jproteome.1c00463

**Published:** 2021-08-23

**Authors:** Sam G. Galvin, Richard G. Kay, Rachel Foreman, Pierre Larraufie, Claire L. Meek, Emma Biggs, Peter Ravn, Lutz Jermutus, Frank Reimann, Fiona M. Gribble

**Affiliations:** † University of Cambridge Metabolic Research Laboratories, WT-MRC Institute of Metabolic Science, Addenbrooke’s Hospital, Hills Road, Cambridge, CB2 0QQ, U.K.; ‡ Research and Early Development Cardiovascular, Renal and Metabolism (CVRM), BioPharmaceuticals R&D, AstraZeneca Ltd., Cambridge, CB21 6GH, U.K.

**Keywords:** pancreatic islets, mass spectrometry, peptidomics, type 2 diabetes, insulin, glucagon

## Abstract

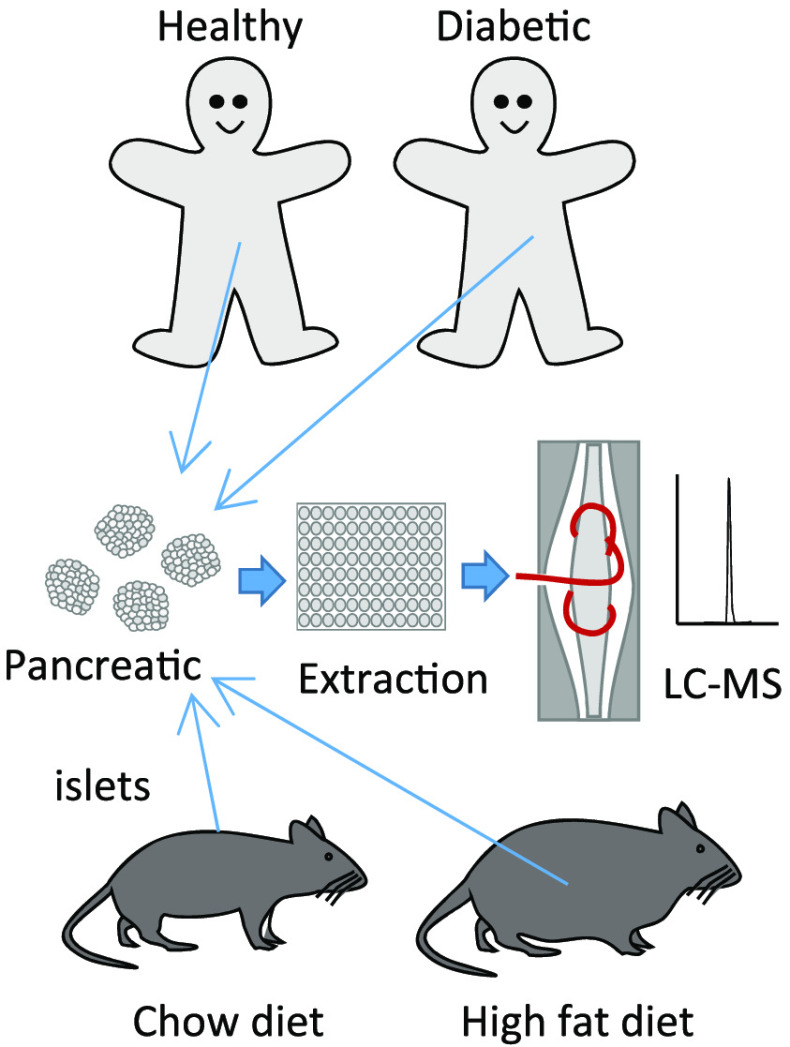

To characterize the impact of metabolic disease on the peptidome of human and mouse pancreatic islets, LC-MS was used to analyze extracts of human and mouse islets, purified mouse alpha, beta, and delta cells, supernatants from mouse islet incubations, and plasma from patients with type 2 diabetes. Islets were obtained from healthy and type 2 diabetic human donors, and mice on chow or high fat diet. All major islet hormones were detected in lysed islets as well as numerous peptides from vesicular proteins including granins and processing enzymes. Glucose-dependent insulinotropic peptide (GIP) was not detectable. High fat diet modestly increased islet content of proinsulin-derived peptides in mice. Human diabetic islets contained increased content of proglucagon-derived peptides at the expense of insulin, but no evident prohormone processing defects. Diabetic plasma, however, contained increased ratios of proinsulin and des-31,32-proinsulin to insulin. Active GLP-1 was detectable in human and mouse islets but 100–1000-fold less abundant than glucagon. LC-MS offers advantages over antibody-based approaches for identifying exact peptide sequences, and revealed a shift toward islet insulin production in high fat fed mice, and toward proglucagon production in type 2 diabetes, with no evidence of systematic defective prohormone processing.

## Introduction

Pancreatic islets secrete multiple biologically active peptide hormones, most notably insulin, glucagon, and somatostatin (SST), but a number of recent studies have highlighted the possibility that they might also secrete the incretin hormones, glucagon-like peptide 1 (GLP-1)^1−6^ and glucose-dependent insulinotropic peptide (GIP).^7,8^ Intraislet release of GLP-1 has been postulated to modulate insulin secretion under conditions such as metabolic stress, although it remains unclear whether it is produced locally at levels sufficient to exert physiologically significant effects on beta cells. The literature is complicated by the use of antibody-based methods to detect and quantify peptide hormones as these are prone to antibody cross-reactivity, but improvements in mass spectrometry methods now allow identification and quantification of exact peptide sequences. This study aimed to identify the exact peptide sequences produced and released from mouse and human islets, and the impact of consuming a high fat diet or development of type 2 diabetes.

Five islet cell populations have been described, including the 3 major cell types: beta cells producing insulin and islet amyloid polypeptide (IAPP), alpha cells producing glucagon and delta cells producing SST-14, together with rarer PP-cells producing pancreatic polypeptide (PPY), and epsilon cells producing ghrelin.^9^ Pancreatic islet development shares common endodermal origins and transcription factor requirements with intestinal enteroendocrine cells, so it is not surprising to find overlap of hormone expression between islets and the gut. Indeed, the intestinal hormone peptide YY (PYY) has been detected in alpha, delta, and gamma cells in mouse but not humans.^10,11^ GIP has been detected as a shortened version in human and mouse islets (GIP(1–30), compared with (1–42) in the gut), although some antibodies against GIP have been questioned due to their propensity to bind to proglucagon derived peptides,^12−14^ and transcriptomic studies failed to find expression of *Gip* in mouse^11^ or human islets.^10^

Proglucagon is processed by prohormone convertase (PC) 2 in islets to release glucagon, and by PC1/3 in the gut to generate bioactive GLP-1(7–37/7–36amide). Longer forms of GLP-1(1–37/1–36amide) have been identified in human and rat pancreas^15^ and are not bioactive against the GLP-1 receptor (GLP1R) but cross-react with many antibodies against GLP-1. Antagonizing GLP1R attenuates glucose-stimulated insulin secretion (GSIS) from human and mouse islets even in the absence of an intestinal source of GLP-1,^4,6,16,17^ suggesting a local islet source of a GLP1R agonist peptide, but this need not be GLP-1, as glucagon itself acts on the GLP-1 receptor, albeit with 50–100-fold lower potency than active GLP-1.^18,19^ Several studies utilizing liquid chromatography coupled to mass spectrometry (LC-MS) have detected active GLP-1 in islets but have not commented on its abundance relative to glucagon.^1,20^

In addition to clarifying controversies around intraislet GLP-1 and GIP, unbiased LC-MS has potential for elucidating how the islet peptidome responds to metabolic stress. Obesity is well-known to increase insulin secretion, and in rodent models causes beta cell hyperplasia.^21^ In type 2 diabetes (T2DM) and diabetic mouse models, there have been reports of beta cell dedifferentiation,^22−24^ increased alpha cell numbers, and islet GLP-1 production.^1,2^

In this study, we used LC-MS to probe the peptidome of human and mouse islets in health and under conditions of obesity and T2DM, and to analyze intraislet production of incretin peptides. Using similar LC-MS peptidomic methods, we have previously identified and quantified endocrine peptides in a variety of tissues, plasma, and cell supernatants.^25−27^

## Methods

Unless otherwise stated, all chemicals were obtained from Sigma-Aldrich (Poole, UK). GLP-1(7–36 amide) and glucagon standards were from Bachem (Bubendorf, Switzerland). Internal standards for GLP-1(7–36 amide) and glucagon were from Cambridge Research Biochemicals (Billingham, UK).^28^

### Mice

All work was conducted in keeping with the Animals (Scientific Procedures) Act 1986 Amendment Regulations of 2012 and approved by the University of Cambridge Animal Welfare and Ethical Review Board under project licenses 70/7824 and PE50F6065. Mice (either gender, if not stated otherwise) were on a C57BL/6 background, bred in-house under SPF conditions and between 10 and 29 weeks old. For the diet-induced obese (DIO) study, 9–15 week old male mice were assigned to 1 of 2 groups; one fed high fat diet (HFD) (60% fat, Research Diets) for 13 weeks and the other standard chow. Fasting blood glucose levels were taken after 6 h fast. Sixty islets from each mouse were isolated and lysed as below.

### Islet Isolation

Mice were sacrificed by cervical dislocation and the pancreas injected with ice-cold Collagenase V (0.75 mg/mL) in HBSS. After digesting the pancreas at 37 °C for 12 min, islets were washed and hand-picked into HBSS with 0.1% BSA (w/v).

### Islet Lysate Peptidomics

Islets were washed in HBSS before lysing in a Protein LoBind Eppendorf with 200 μL 6 mol/L guanidine hydrochloride (GuHCl). Three freeze thaw cycles were carried out to aid cell lysis. Proteins were precipitated by adding 800 μL of 80% ACN (v/v) and centrifuging at 4 °C for 5 min at 12 000*g*. The aqueous lower phase containing the peptides was collected, dried in a centrifugal evaporator overnight and stored at −70 °C.

### Isolation of Islet Cell Populations

Beta, alpha, and delta cell populations were purified using an Influx Cell Sorter (BD Biosciences, Franklin Lakes, NJ) at the Cambridge Institute for Metabolic Research flow cytometry group. Beta and alpha cells were purified from islets of Glu-GFP mice and delta cells from Sst-Cre/EYFP mice, as described previously.^11^ Cells were sorted into 200 μL 6 mol/L GuHCl, then treated as described for islet lysates.

### Islet Secretion Assays

Fresh islets were incubated at 37 °C in Kreb’s Ringer Buffer (KRB (mmol/L); 129 NaCl, 5 NaHCO_3_, 4.8 KCl, 1.2 KH_2_PO_4_, 1.2 MgSO_4_, 10 HEPES, 2.5 CaCl_2_ with 0.05% BSA (w/v) for 1 h. Islets were transferred to Protein LoBind Eppendorf tubes with 300 μL of prewarmed KRB containing stimuli detailed in figure legends. Tubes were incubated at 37 °C for 45 min, then 270 μL of supernatant was removed and snap frozen. Nine supernatants were pooled for LC-MS analysis.

### Human Islet Study

Ethical approval was obtained from University of Cambridge Human Biology Research Ethics Committee (#HBREC.2019.38). Human islets for research were provided by the Alberta Diabetes Institute IsletCore at the University of Alberta in Edmonton (www.isletcore.ca) with the assistance of the Human Organ Procurement and Exchange (HOPE) program, Trillium Gift of Life Network (TGLN) and other Canadian organ procurement organizations. Islet isolation was approved by the Human Research Ethics Board at the University of Alberta (Pro00013094). All donors’ families gave informed consent for the use of pancreatic tissue in research. Donor characteristics (mean ± st. dev.) are Controls (6 male, 3 female), age 50 ± 5 years, BMI 30 ± 3 kg/m^2^; T2DM (3 male, 4 female), age 52 ± 7 years, BMI 28 ± 4 kg/m^2^, HbA1c 7.3 ± 1.2%, (1 diet-controlled, 4 on metformin, 1 on other oral antihyperglycemic agent, 1 on insulin). Measurements of islet insulin content and Islet Particle Index (a measure of islet size), generated by the Alberta Islet Core at the time of tissue collection, were not significantly different between the T2DM and control groups. Anonymized, snap frozen human islets (2000 islet equivalents (IEQ) per donor, i.e., the standardized equivalent of 2000 islets of diameter 150 μm) were thawed, washed 3× with HBSS, spun at 200*g* for 5 min at 4 °C, and supernatants discarded. Islets were lysed in 250 μL of 6 mol/L GuHCl with 3 freeze thaw cycles, and proteins precipitated as above.

### Preparation of Standard Curves

Calibration curves for glucagon and GLP-1(7–36amide) were prepared in matrix comprising mouse pancreatic acinar tissue from which visible islets had been removed, treated with GuHCl and ACN, as above. Internal standards for glucagon and GLP-1(7–36amide) were spiked into calibration standards and islet lysates.

### Solid Phase Extraction, Reduction, and Alkylation

Solid phase extraction (SPE), reduction and alkylation, were performed as described previously.^29^ Cellular lysates were reconstituted in 0.1% FA (v/v) and supernatants acidified with formic acid to a final percentage of 0.1% (v/v). Samples were extracted on an Oasis PRiME HLB μElution plate (Waters, Milford, MA). Only cellular lysates were reduced and alkylated. Supernatants were run immediately after SPE.

### Nano LC-MS

For detailed methods on columns, source settings, gradient details, and database searching see ref (29). Briefly, samples were analyzed on a Thermo Fisher UltiMate 3000 Nano LC system coupled to a Q Exactive Plus Orbitrap mass spectrometer (ThermoScientific, San Jose, USA) using electrospray ionization in positive mode. Method run time was 130 min with a full MS scan on ions between 400 and 1600 *m*/*z* prior to a MS/MS scan 10 top ions per scan. Product ion scans were used to monitor for specific ions given in Supplementary Table S1. Data files were searched against the mouse SwissProt database (downloaded 26/10/2017) using PEAKS v8.5 software (Waterloo, Ontario, Canada). To quantify data acquired by product ion scans, Xcalibur v4.3.73.11 (Thermo Fisher Scientific) was used to integrate area under the curve on the chromatogram.

### Human Plasma

Stored human plasma from the placebo arm of a previous study^30^ in which healthy volunteers and patients with type 2 diabetes received a 75 g oral glucose tolerance test, was analyzed by LC-MS to measure insulin, proinsulin and des 31–32 proinsulin. Samples were extracted using well established methods^31^ and analyzed on a microflow LC system, coupled to a HSS T3 ionKey (Waters) on the TQ-XS spectrometer. Ten μL of sample was injected onto a trap column at 15 μL/min for a 3 min load, with mobile phases set to 90%A (0.1% formic acid (aq)) and 10% B (0.1% formic acid (acetonitrile)). The ionKey column was set at 45 °C and the analytes were separated over a 13 min gradient from 10% to 55% B, at a flow rate of 3 μL/min. The column was flushed for 3 min at 85% B before returning to initial conditions, resulting in an overall run time of 20 min. Targeted SRM transitions were set up based on parent and precursor ion fragments for each peptide (Supplementary Table S2). Peptide peak areas were quantified using MassLynx v4.2 (Waters) and normalized as peak area ratio against an internal standard, bovine insulin.

### Data Analysis

Data visualization and statistical analysis were carried out using RStudio (v1.3) and R (v4.0.2). When peptidomic differences between one or more groups needed to be assessed, the outputs of PEAKS database searches were obtained and analyzed in R. To control for multiple comparisons, *P* values were adjusted using a permutation-based method using Perseus (Max Planck Institute of Biochemistry, v1.6.14.0).

## Results

### Peptidomics of Human and Mouse Islets

**Figure 1:**
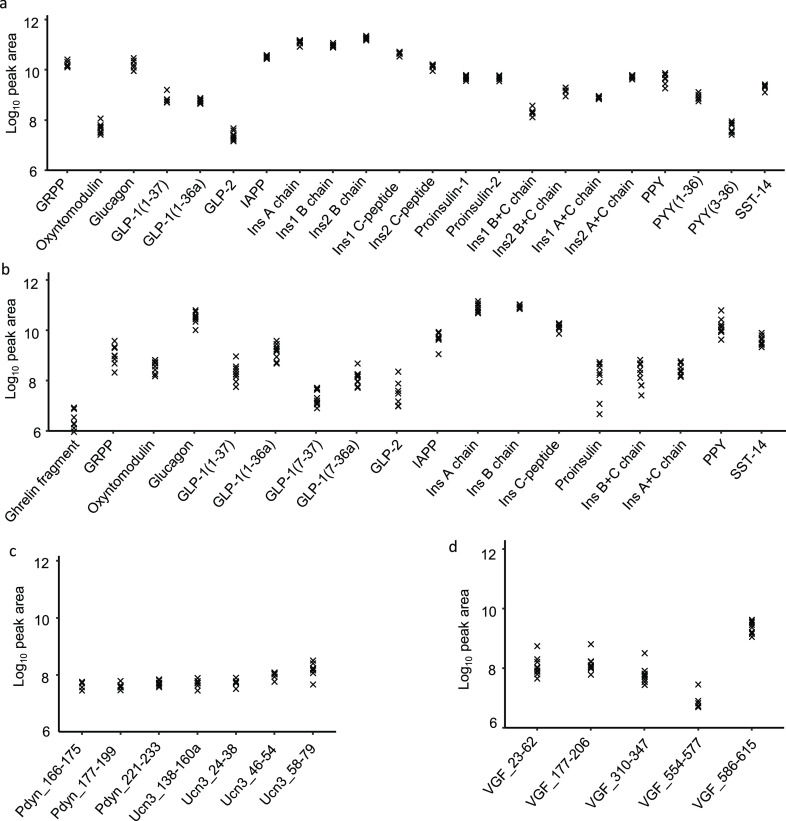
Overview of peptides produced from prohormones in mouse and human islets. Quantification of peptides derived from classical islet prohormones by DDA in mouse islets (a), and human islets (b). Peptides derived from prourocortin-3 and proenkephalin-B in mouse islets (c) and peptides derived from proVGF in human islets (d). If peptides do not have an assigned name in the literature then peptides are named for their gene of origin as well as their position on that gene; e.g., Pdyn_177–199 originates from Pdyn and spans amino acids 177–199. Mouse data from 7 mice. Human data from 9 individuals. N.B. The A chains of insulin-1 an insulin-2 are identical and so only 1 peptide is displayed for the insulin A chain.

The major islet hormones—insulin, glucagon, SST, PPY, and IAPP—were detected by LC-MS/MS in extracts of lysed human and mouse islets (Figure 1a,b). Using data dependent acquisition (DDA), we also detected a range of other peptides, including proinsulin, C-peptide, and partially processed insulins, as well as GRPP (the N-terminus of proglucagon) and inactive forms of GLP-1 (1–36amide and 1–37). Active GLP-1 was detectable only in the human samples with this method (Figure 1b). An N-terminal fragment of ghrelin (GHRL_24–37) was detected in human islets, but full length and acylated versions of ghrelin were not detectable in either species. In mouse islets we detected PYY and fragments of prourocortin-3 and proenkephalin-B (alpha-neoendorphin and rimorphin)^32,33^ (Figure 1c), and in human islets we found peptides from neurosecretory protein VGF (Figure 1d). We also detected a number of peptides derived from granin proteins and vesicular processing enzymes (Supplementary Figure S1).

### Peptidomics of Purified Alpha, Beta, and Delta Cells

**Figure 2:**
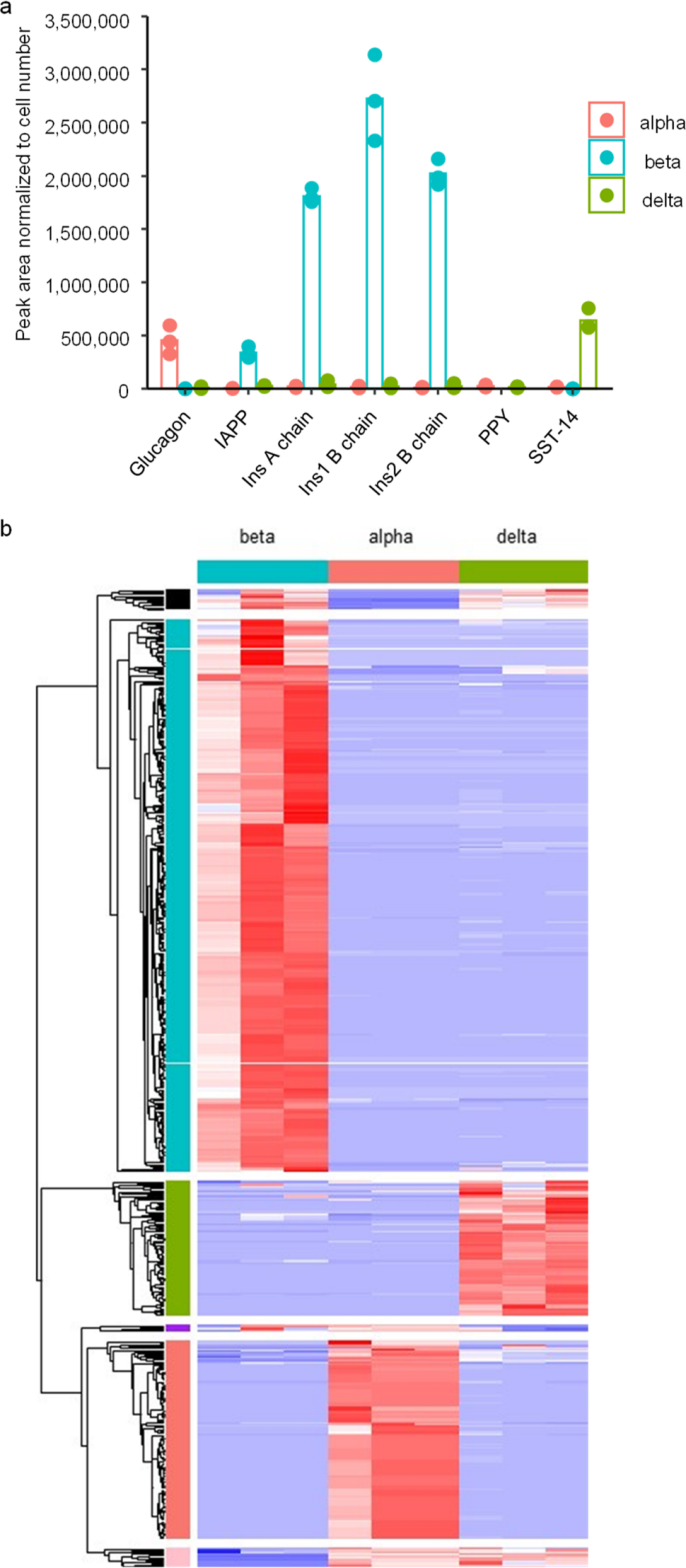
Peptidomics of FACS purified mouse alpha beta and delta cells. (a) Abundance of major islet hormones in the three FACS purified islet cell populations. (b) Heatmap of all peptides matched through Peaks database searching in the 3 purified cell populations. In all plots, the peak area of each peptide was normalized to the number of cells in that sample to account for differences in cell numbers. For alpha and beta FACS purified cells, 3 separate samples were obtained from 3 individual mice, whereas delta cells from 3 pairs of mice were pooled to obtain 3 samples.

We determined the cellular origin of the different peptides by analyzing FACS-purified mouse alpha, beta and delta cells. Across all islet cell samples, 999 peptides were matched by PEAKS database searching, of which 559 were detectable in at least 2 samples. Peptides from proinsulin, proglucagon and proSST separated across the beta, alpha and delta cells respectively, as expected (Figure 2a and Supplementary Table S3). Pancreatic polypeptide (PPY) was detected in alpha and delta cells, although our method of cell separation may have excluded collection of a cell type specifically expressing PPY. PYY was mostly found in delta cells. Peptides from urocortin 3 and proenkephalin-B were found at highest levels in beta cells, together with IAPP (Supplementary Table S3). Most peptides were predominantly identified in a single cell population (Figure 2b and Supplementary Table S3).

### Intraislet GLP-1 and GIP

**Figure 3:**
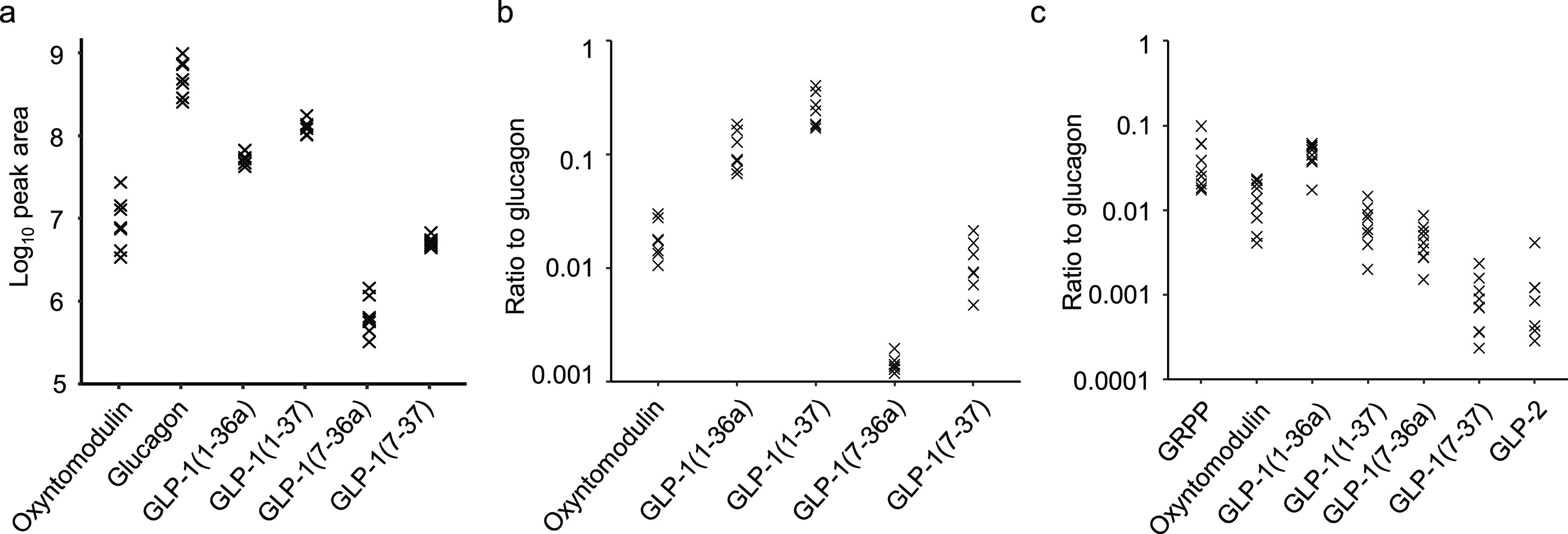
Proglucagon derived peptides. (a,b) Peptides in mouse islets quantified by monitoring for specific product ions after fragmenting precursor ions specific to each peptide. (a) Quantification of proglucagon derived peptides in islet lysates from 7 mice. Each sample contained 60 islets. (b) Ratios of proglucagon derived peptides to glucagon in mouse islets. (c) Ratios of proglucagon derived peptides to glucagon, monitored by DDA, in human islets.

Although active forms of GLP-1(7–36amide and 7–37) were readily detectable in human islets, their peak areas were substantially smaller than those of either glucagon or N-terminally extended inactive GLP-1(1–36 amide and 1–37) (Figure 1b and 3c). From standard curves for glucagon and GLP-1(7–36amide), we estimated that human islets contained 1.2 ± 0.4 pg/IEQ (mean ± SEM, *n* = 9) of GLP-1(7–36amide), whereas glucagon concentrations were above the highest calibration standard, equivalent to >150 pg/IEQ, with an estimated value of ∼370 pg/IEQ (Supplementary Figure S2). From both the calibrated results and the peak areas shown in Figure 1 and 3c, the glucagon:GLP-1 ratio in human islets was estimated to be >100:1, and likely closer to 300:1.

In mouse islet lysates, GLP-1(7–36amide and 7–37) could be detected when we used product ion scans to monitor for their fragments, even though they had not been detectable in DDA mode (Supplementary Figure S3). Both b and y ions matching GLP-1 were identified from a peak that coeluted with a standard for GLP-1(7–36 amide), providing compelling evidence for the intraislet production of GLP-1(7–36 amide) in mouse as well as humans. Using the product ion scanning approach, we again estimated the relative abundance of different proglucagon-derived peptides (Figure 3a). Using standard curves, glucagon was quantifiable at 3900 ± 400 pg/islet (mean ± SEM, *n* = 7) whereas GLP-1(7–36 amide) and GLP-1(7–37) were below the lower limit of quantification, and likely therefore <0.8 pg/islet. Both the calibrated data and peak areas suggest a glucagon:GLP-1 ratio of >1000:1 in mouse islets (Figure 3b and Supplementary Figure S2).

Product ion monitoring was also used to search for proGIP derived peptides in mouse islets as none were detected using DDA. We were unable to detect GIP(1–42) (the intestinal form), C-terminally truncated GIP(1–30) (previously described in islets^12^), or a peptide from the N-terminus of proGIP (Gip_22–43) that we can identify robustly in homogenized mouse duodenal tissue (Supplementary Figure S4–S6). GIP was also not detectable in human islets by DDA analysis.

### Peptidomics of Lean versus Diet-Induced Obese (DIO) Mouse Islets

**Figure 4:**
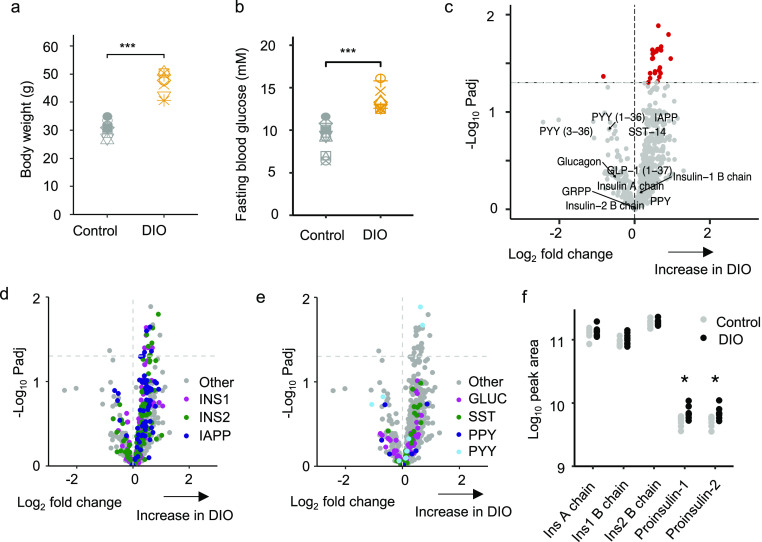
Peptidomic comparison of islets from DIO mice and to lean controls. Body weights (a) and fasting blood glucose levels (b) of DIO mice vs lean controls. (c–e) Volcano plots displaying log2 fold change vs −log10 of the adjusted p value for each peptide. Horizontal dotted line indicates significance threshold of *p* = 0.05. A positive log2 fold change indicates an increase in DIO mice. 718 *t* tests were performed to analyze for significant differences in peptides matched between the groups with a permeation-based method used to adjust for multiple comparisons. In (d,e), peptides from different prohormones are colored in each plot: IAPP and INS derived peptides in (d). GLUC, SMS, PPY, and PYY derived peptides in (e). (f) Peak area of processed insulin-1 and -2 chains in addition to proinsulin-1 and -2. Statistical comparison made using unpaired *t* test without adjustments for multiple comparisons. *p < 0.05. *n* = 7 for control group and *n* = 8 for DIO group.

To assess the effect of obesity on the islet peptidome, mice were fed a high-fat diet for 13 weeks, at the end of which they displayed higher body masses and fasting blood glucose than chow-fed controls (Figure 4a,b). Size-matched islets from both groups were compared by LC-MS/MS, with the results depicted in volcano plots (Figure 4c–e). Fully processed insulin-1, insulin-2, glucagon, SST, and PPY were not significantly different between the groups. However, a number of other fragments from proinsulin and proIAPP were significantly increased in the islets from DIO mice (Figure 4d), as well as 2 peptides from proPYY (Figure 4e). Peptides from granins and processing enzymes were largely unchanged (Supplementary Figure S7a–c and Supplementary Table S4). Manual quantification of proinsulins-1 and -2, which are too long to be matched automatically by the PEAKS software, revealed a significant increase in proinsulin-1 and -2 in the DIO islets (Figure 4f).

### Peptidomics of Type 2 Diabetic Islets and Plasma

**Figure 5:**
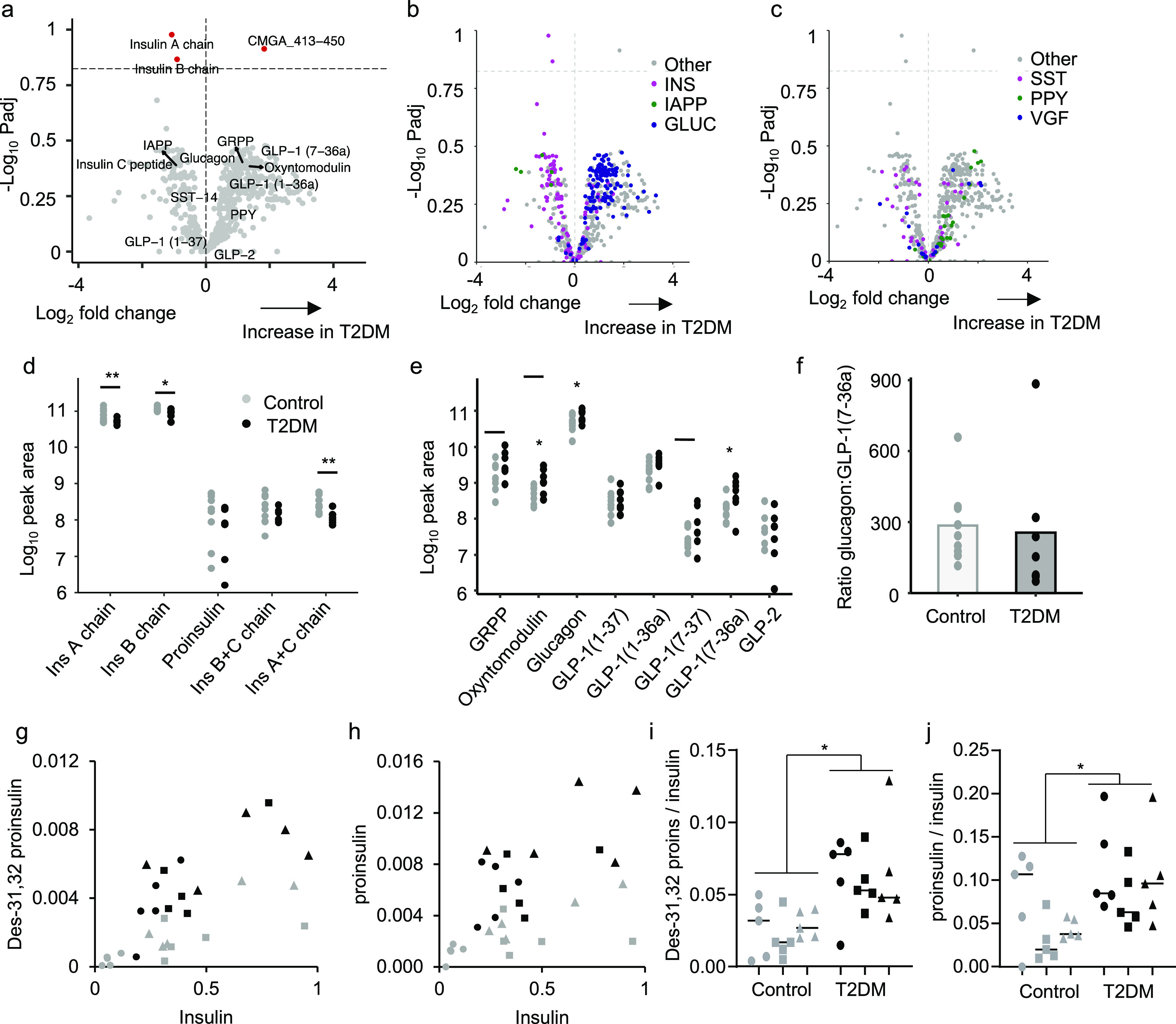
Peptidomic comparison of human islets from type 2 diabetic individuals compared to nondiabetic controls. (a) Volcano plot displaying log2 fold change vs −log10 of the adjusted *p* value for each peptide. Horizontal dotted lines indicates *p* = 0.15. A positive log2 fold change indicates an increase in T2DM. (b,c) Volcano plots displaying in different colors individual peptides derived from proIAPP, proinsulin, and proglucagon (b), and from prosomatostatin, proPPY, and proVGF (c). (d,e) Manually quantified peak areas of processed insulin chains and proinsulin (d), and products of proglucagon processing (e). (f) Ratio of peak area of glucagon:GLP-1(7–36amide) in all samples. Statistical comparison made using unpaired *t* test without adjustments for multiple comparisons. *p < 0.05. For a–f, *n* = 9 for the control group and *n* = 7 for T2DM group. Donors were excluded from the analysis if the samples failed to load properly onto the SPE sorbent. (g,h) Plasma des-31,32 proinsulin (g) and proinsulin (h) plotted against insulin for control (gray) and T2DM (black) subjects in the fasting state (circles), and 30 min (squares) or 90 min (triangles) after a 75 g oral glucose tolerance test. Values represent peak area ratios. (i,j) Ratios of data shown in (g,h). *p < 0.05 between controls and T2DM by 2-way ANOVA.

To investigate the effects of type 2 diabetes on the islet peptidome, we compared nondiabetic and diabetic islets (Figure 5a–c, Supplementary Figure S7d,e and Supplementary Table S5). No individual peptides were significantly altered in diabetes when *p*-values were adjusted for multiple comparisons. However, multiple peptides from proinsulin and IAPP clustered on the “reduced in T2DM” side of the volcano plot, whereas peptides from proglucagon clustered on the “increased in T2DM” side (Figure 5b). Somatostatin-derived peptides exhibited no clear divide, and peptides from PPY were mostly increased in diabetes (Figure 5c).

Manual quantification confirmed significant reductions in insulin A and B chains but not proinsulin in the T2DM group (Figure 5d) and increases in glucagon and GLP-1(7–36amide) (Figure 5e). Using a calibration line, GLP-1(7–36amide) increased from 1.2 ± 0.4 pg/IEQ (mean ± SEM; *n* = 9 nondiabetic donors) to 3.6 ± 0.9 pg/IEQ (*n* = 7 diabetic donors, *p* = 0.025). However, although glucagon measurements were above the top calibration standard, the peak area ratio for glucagon increased in parallel, and the ratio of peak areas for glucagon:GLP-1 was not significantly different between the groups (Figure 5f), suggesting an overall increase in proglucagon biosynthesis (which could reflect either a change in alpha cell number, or proglucagon biosynthesis per cell) rather than a change in processing.

Proinsulin products were also measured by LC-MS/MS in the plasma of control and diabetic volunteers. Both before and after an oral glucose challenge, we observed elevated circulating levels of proinsulin and des-31,32 proinsulin in the diabetic group, which were proportionally increased following glucose ingestion, suggesting that incompletely processed proinsulin products exhibit glucose-sensitive secretion and are coreleased with insulin following glucose challenge (Figure 5g,h).

### Secretory Patterns from Mouse Islets

**Figure 6:**
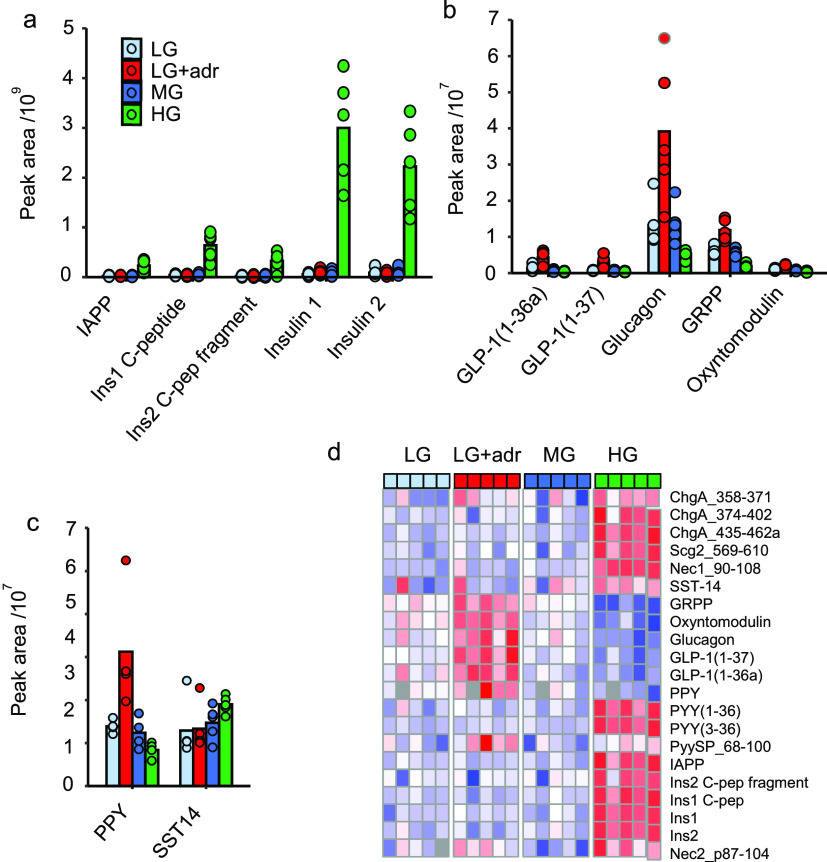
Peptidomics of secretions from mouse islets. (a–c) Peak areas of peptides in supernatants of islets cultured in low, medium, or high glucose, or low glucose + adrenaline. IAPP and insulin-derived peptides (a), proglucagon-derived peptides (b), PPY and SST-14 (c). Individual data points are displayed in each plot along with the mean. Conditions were as follows; LG = 1 mmol/L glucose, LG + Adr = 1 mmol/L glucose + 5 μmol/L adrenaline, MG = 6 mmol/L glucose, HG = 16.7 mmol/L glucose. *n* = 5 with each data point representing pooled supernatants from 9 tubes each containing 15 islets. Islets obtained from 15 mice. (d) Heatmap of mouse islet secretion peptidomics. Data presented as Z-score of each row. Gray squares within the heatmap indicate where peptides were not detectable. PyySP_68-100 is a peptide from a splice variant of *Pyy* which has 2 amino acids inserted into the C-terminus of the propeptide. It was quantified in lieu of Pyy_68-98 which was undetectable.

Mouse islets were incubated with low (1 mmol/L), medium (6 mmol/L) or high (16.7 mmol/L) glucose, or low glucose + adrenaline (10 μmol/L), to trigger differential secretion from alpha, beta and delta cells. Proglucagon-derived peptides and PPY showed higher secretion in the low glucose + adrenaline condition, whereas proinsulin-derived peptides, IAPP and SST exhibited highest secretion in high glucose (Figure 6). Active GLP-1 and peptides from proenkephalin-B and urocortin 3 were not detectable in islet supernatants.

## Discussion

This study presents a detailed analysis of the human and mouse islet peptidome with a focus on peptides derived from secretory granules, together with corresponding measurements in murine obesity and human T2DM. Mostly we applied a semiquantitative approach that enabled comparisons between the same peptide in different specimens, but did not generate exact concentrations. For glucagon and GLP-1, the additional use of calibration standards allowed assessments of actual peptide contents. We are not able to draw conclusions about the presence or absence of peptides that were undetectable using this methodology and for which we did not include individual peptide standards, because different peptides are not uniform in their behavior during the extraction steps and LC-MS analysis.

In addition to peptides from proinsulin, proglucagon, and prosomatostatin, we identified some rarer peptides originating from VGF in human islets, and from PYY, prourocortin-3 and proenkephalin-B in mouse islets. Proenkephalin-B is primarily expressed in the brain^34^ where it is processed to multiple opioid receptor agonists such as α-neoendorphin and rimorphin,^32,33^ but has not been described previously in islets. As opioid receptors were not detected in human or mouse islets by RNA sequencing,^10,11^ it seems unlikely that proenkephalin-products are a key player in intraislet cross talk. Urocortin-3 was described previously in pancreatic beta cells^35^ and confirmed here by LC-MS.

Mirroring reports that *Gip* mRNA is not detectable in islets^10,11^ and absence of islet Cre-reporter activity in GIP-Cre mice,^36^ we could not detect proGIP derived peptides in mouse or human islets despite using targeted product ion scans to monitor for 3 individual proGIP peptides that are readily detectable in duodenum.^25^ We conclude it is highly unlikely that human or mouse islets produce GIP.

Estimates for islet glucagon content measured by LC-MS were similar to those measured previously by ELISA.^37−39^ Active GLP-1(7–36 amide and 7–37) was detectable by LC-MS in mouse and human islets, but at 400 to 1000-fold lower levels than glucagon. Very low levels and secretion of active GLP-1 relative to glucagon were previously reported in mouse islets,^4,16^ but other studies using antibody-based approaches that are less able to discriminate GLP-1(7–37/36amide) from GLP-1(1–37/36amide) have calculated islet GLP-1 production to be much higher.^2,4,40^ Our LC-MS approach readily detected N-terminally extended GLP-1(1–37/1–36amide) in human and mouse islets, with peak areas ∼10-fold higher than corresponding GLP-1(7–37/36amide) forms. Despite the relatively low production of active GLP-1 by pancreatic alpha cells, a number of studies have concluded that intraislet production of proglucagon-derived peptides influences insulin secretion through beta cell GLP1R.^4,6,16,40^ The difference in potency between GLP-1(7–36amide) and glucagon on GLP1R has been estimated at 50 to 400-fold,^16,19,41,42^ so our finding of 300–1000 times more glucagon than active GLP-1 in islets would favor glucagon as the local dominant agonist on beta cell GLP1R. All detectable proglucagon fragments were released in parallel in secretion experiments, exhibiting lower release at high glucose, suggesting that signaling from alpha to beta cells via GLP1R might diminish in importance following a simple rise in plasma glucose concentration. However, GLP1R-dependent cross-talk between alpha and beta cells might be higher in situations when alpha cells are simultaneously activated, such as in the postprandial state when they are directly stimulated by amino acids and/or gut-derived GIP, as also suggested by a recent study.^43^

In islets from DIO mice we observed an increase in the abundance of proIAPP, proinsulin-1 and -2 derived peptides, consistent with reports of β-cell hyperplasia in similar models.^44−46^ However, as we deliberately matched islet sizes between the control and DIO group, our analysis would have excluded larger islets with beta cell hyperplasia, reducing our ability to quantify differences in islet insulin content. No other significant peptidomic changes were seen in DIO mouse islets, supporting an RNA-sequencing based approach which similarly did not find major transcriptomic differences in alpha cells between DIO and lean mice.^47^

To our surprise, we detected only limited peptidomic changes in islets from diabetic human donors. However, as islets had a mean culture time of 43 h in 5.5 mmol/L glucose and cold ischemia time of 14 h prior to freezing, this may have been sufficient to reverse effects of hyperglycemic stress encountered in vivo. As we only analyzed samples from 9 control and 7 diabetic donors, across a spectrum of diabetes severity, this study is not powered to correlate peptidomic changes with patient phenotypes, or identify changes in specific subgroups. Overall, diabetic islets exhibited a global reduction in peptides from proinsulin and IAPP and a corresponding increase in peptides from proglucagon, with no evident change in SST. This mirrors results from a previous study measuring insulin, glucagon, and SST contents in pancreatic tissue from T2DM and nondiabetic donors, which reported lower insulin content per gram of tissue in the diabetic group and not significantly altered glucagon or SST, although reductions in the total pancreatic contents of insulin and SST content were evident when the smaller overall weight of T2DM pancreas was taken into account.^48^ Contrary to our expectations, based on previous reports that T2DM is associated with increased circulating proinsulin levels^49,50^ and increased islet GLP-1 production,^1^ we found no evidence of substantially altered proinsulin or proglucagon processing in T2DM islets. Some studies have suggested that beta cells dedifferentiate in diabetes, taking on partial alpha cell phenotypes and expressing *GCG* together with *PC1/3*.^22,51^ In theory, this could generate cells capable of producing GLP-1(7–36amide) from proglucagon, potentially explaining previous reports of increased islet GLP-1 generation in diabetes. Although manual quantification of our LC-MS data revealed a significant increase in active GLP-1 in diabetic islets, this was mirrored by an increase in glucagon with no change in the glucagon:GLP-1 ratio, arguing against a major alteration in proglucagon processing.

Despite detecting no shift in insulin processing in diabetic islets, in plasma from a separate group of diabetic volunteers, we noticed an increase in the ratio of proinsulin and des-31,32 proinsulin to mature insulin, compared with healthy controls, supporting a number of previous studies employing immunoassays.^49,50^ Proinsulin and des-31,32 proinsulin increased proportionally with insulin following glucose ingestion, suggesting that both peptides are released in parallel in vivo. The finding of increased plasma proinsulin and des-31,32 proinsulin in plasma from the diabetic group, without corresponding increases in partially processed insulin fragments in the islets, is compatible with the idea that islets in type 2 diabetes release more immature vesicles containing incompletely processed proinsulin.

In conclusion, this analysis has identified the spectrum of peptides produced by human and mouse islets in health and metabolic disease, including post-translational modifications and exact peptide sequences. While we could detect active GLP-1 in both human and mouse islets, levels were 100–1000 fold lower than glucagon, suggesting that the activity of glucagon on beta cell GLP1R would overcome any effect of local GLP-1 production. Locally released GLP1R agonist peptides could contribute to postprandial insulin release, particularly when alpha cells are stimulated by elevated levels of intestinal GIP and amino acids.

## Supporting Information Available

Table S1: Precursor ions selected for product ion scans; Table S2: Product, precursor, collision energies, and dwell times for peptides monitored on triple quadrupole mass spectrometer; Figure S1: Nonclassical islet peptides detected in mouse and human islets; Figure S2: Standard curves for GLP-1(7–36amide) and glucagon; Figure S3: GLP-1(7–36amide) in mouse islets; Figure S4: Searching for the N-terminal propeptide of proGip (Gip 22–43) in mouse islets; Figure S5: Searching for GIP (1–30) in mouse islets; Figure S6: Searching for GIP (1–42) in mouse islets; Figure S7: Peptidomic comparison of islets from DIO mice and humans with type 2 diabetes (PDF)
Table 3: Peptidomics of murine alpha, beta, and delta cells (XLSX)
Table 4: Peptidomic differences between islets from mice fed on chow or high fat diet (XLSX)
Table 5: Peptidomic differences between nondiabetic control and type 2 diabetic donor islets (XLSX)

## Notes

The mass spectrometry proteomics data have been deposited to the ProteomeXchange Consortium via the PRIDE partner repository with the data set identifier PXD026095 and 10.6019/PXD026095.

## Abbreviations

ACN: acetonitrile
DIO: diet-induced obese
GIP: glucose-dependent insulinotropic peptide
GLP-1: glucagon like peptide-1
GLP1R: GLP-1 receptor
GuHCl: guanidine hydrochloride
HFD: high fat diet
IAPP: islet amyloid polypeptide
LC-MS: liquid chromatography coupled to mass spectrometry
PC: prohormone convertase
PPY: pancreatic polypeptide
PYY: peptide YY
SST-14: somatostatin-14.
