# Supplementary material for: The Human and Mouse Islet Peptidome: Effects of Obesity and Type 2 Diabetes, and Assessment of Intraislet Production of Glucagon-like Peptide-1
Source: J Proteome Res. 2021 Aug 23;20(9):4507–17. doi: 10.1021/acs.jproteome.1c00463 (PMC8419866; doi:10.1021/acs.jproteome.1c00463)
Supplement: Supplementary file 1 — pr1c00463_si_001.pdf [file pr1c00463_si_001.pdf]

The human and mouse islet peptidome: effects of obesity and type 2 diabetes, and assessment of intra-islet production of glucagon-like peptide-1

Sam G. Galvin[1], Richard G. Kay[1], Rachel Foreman[1], Pierre Larraufie[1], Claire L Meek[1], Emma Biggs[1], Peter Ravn[2], Lutz Jermutus[2], Frank Reimann*[1], Fiona M. Gribble*[1]

[1] University of Cambridge Metabolic Research Laboratories, WT-MRC Institute of Metabolic Science, Addenbrooke's Hospital, Hills Road, Cambridge, CB2 0QQ, UK

[2] Research and Early Development Cardiovascular, Renal and Metabolism (CVRM), BioPharmaceuticals R&D, AstraZeneca Ltd, Cambridge, CB21 6GH, UK

* Joint corresponding authors

Supporting information

Suppl table 1: Precursor ions selected for product ion scans.

Suppl table 2: Product, precursor, collision energies and dwell times for peptides monitored on triple quadrupole mass spectrometer.

Suppl fig 1: Non-classical islet peptides detected in mouse and human islets

Suppl fig 2: Standard curves for GLP-1 (7-36 amide) and glucagon

Suppl fig 3: GLP-1(7-36 amide) in mouse islets

Suppl fig 4: Searching for the N-terminal propeptide of proGip (Gip 22-43) in mouse islets.

Suppl fig 5: Searching for GIP (1-30) in mouse islets.

Suppl fig 6: Searching for GIP (1-42) in mouse islets

Suppl fig 7: : Peptidomic comparison of islets from DIO mice and humans with type 2 diabetes.

Peptide	Precursor ion (m/z)	Normalised collision energy
Oxyntomodulin	636.600	26
Glucagon	871.600	26
GLP-1 (1-37)	695.840	26
GLP-1 (1-36 amide)	686.170	26
GLP-1 (7-37)	839.600	26
GLP-1 (7-36 amide)	825.400	26
GIP (1-42)	834.422	26
GIP (1-30)	895.220	26
Gip_22-43	516.070	26

Supplementary table S1: Precursor ions selected for product ion scans. The isolation width for each precursor ion was set at 4 *m/z.*

Peptide	Precursor (m/z)	Product (m/z)	CollisionEnergy	Dwelltime (ms)
Human insulin	1162.2	226.3	35	0.025
Human des 31,32 proinsulin	1300.0	785.36	25	0.025
Human proinsulin	1342.1	219.1	35	0.025
Bovine insulin (internal standard)	956.3	1120.8	22	0.025

Supplementary table S2: Product, precursor, collision energies and dwell times for peptides monitored on triple quadrupole mass spectrometer.

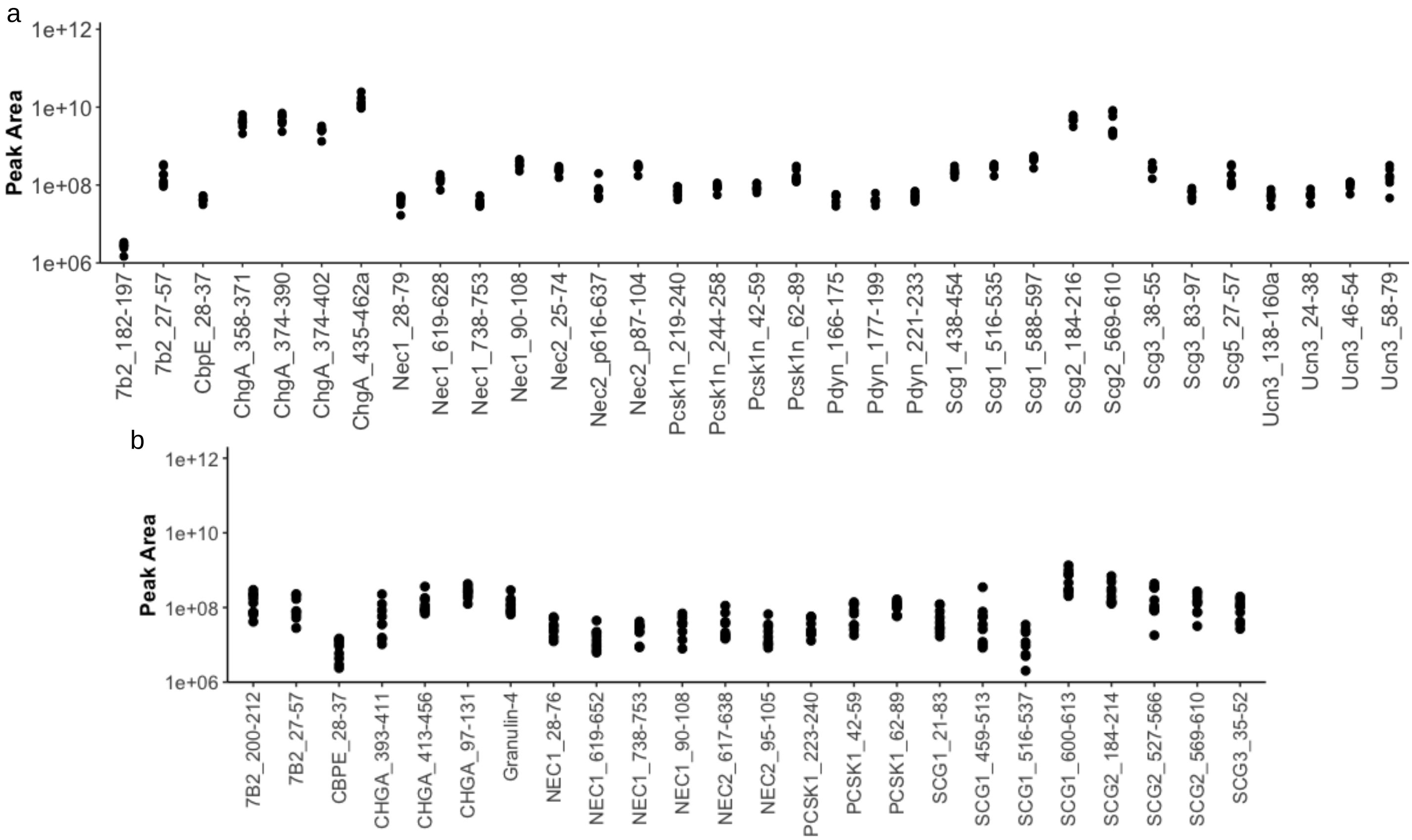

Supplementary figure 1: **Non-classical islet peptides detected in mouse (a) and human (b) islets.** If peptides do not have an assigned name in the literature then peptides are named for their gene of origin as well as their position on that gene. E.g. 7b2_182-197 originates from *7b2* and spans amino acids 182-197.

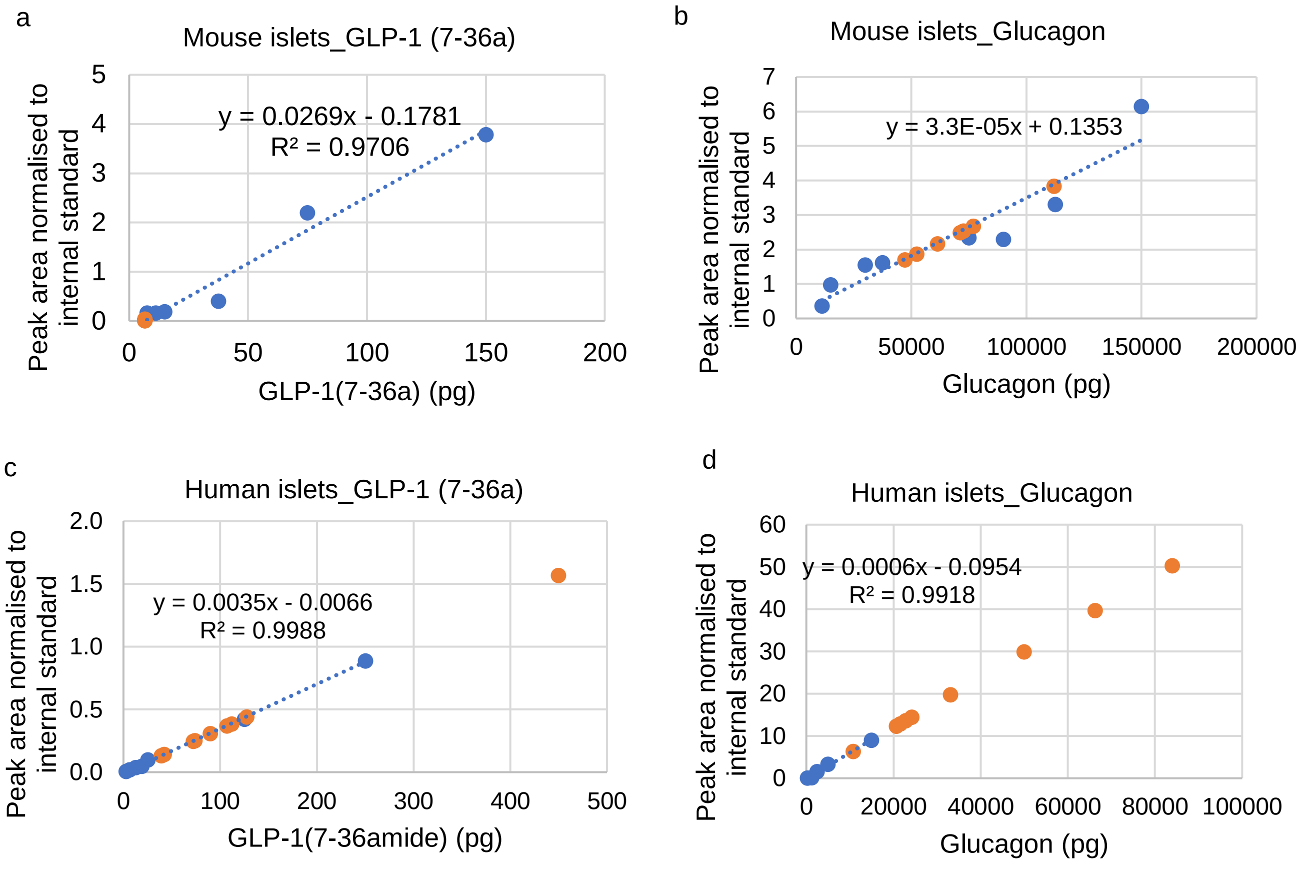

Supplementary figure 2:
a,b. Standard curves for GLP-1 (7-36 amide) (a) and glucagon (b) with mouse islet samples overlaid.
c,d. Standard curves for GLP-1 (7-36 amide (c) and glucagon (d) with human islet samples overlaid.
Blue: standards. Orange: samples. Linear fit to standards shown by dotted lines

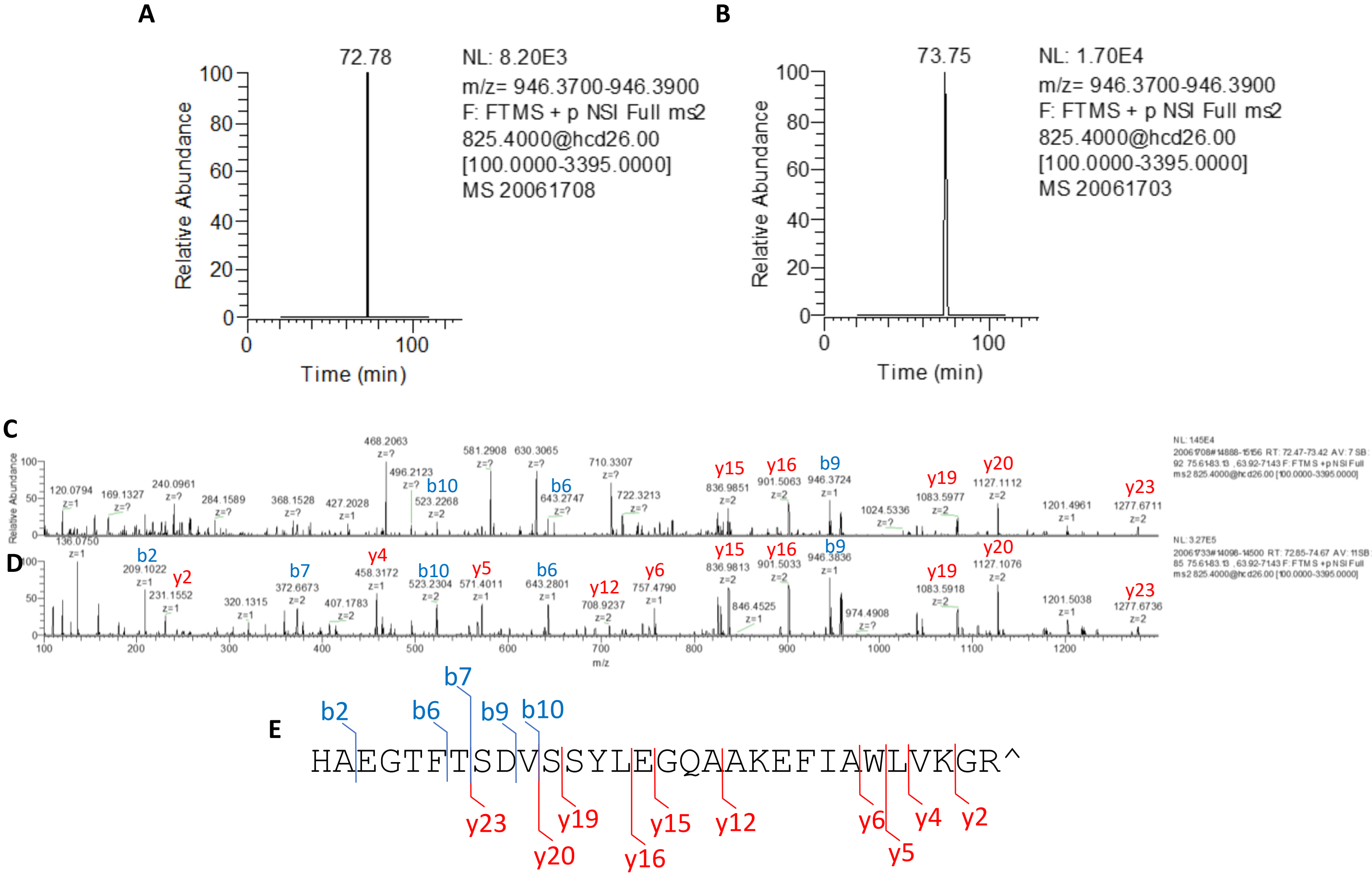

Supplementary figure 3: GLP-1(7-36 amide) in mouse islets.

a,b. Extracted ion chromatograms from a MS/MS method monitoring for fragments of endogenous GLP-1 (7-36 amide) in mouse islets (a) and a GLP-1(7-36 amide) standard (b). Peaks can be seen at 72.78 and 73.75 min in both traces which correspond to GLP-1(7-36 amide).

c. Product ion spectrums corresponding peak at 72.78 min in (a)

d. Product ion spectrums corresponding peak at 73.75 min in (b).

e. Sequence of GLP-1(7-36 amide). ^ represents C-terminal amidation. The masses of certain ions shown in (c) and (d) correspond to the masses of b and y ions from GLP-1(7-36 amide) and have been annotated.

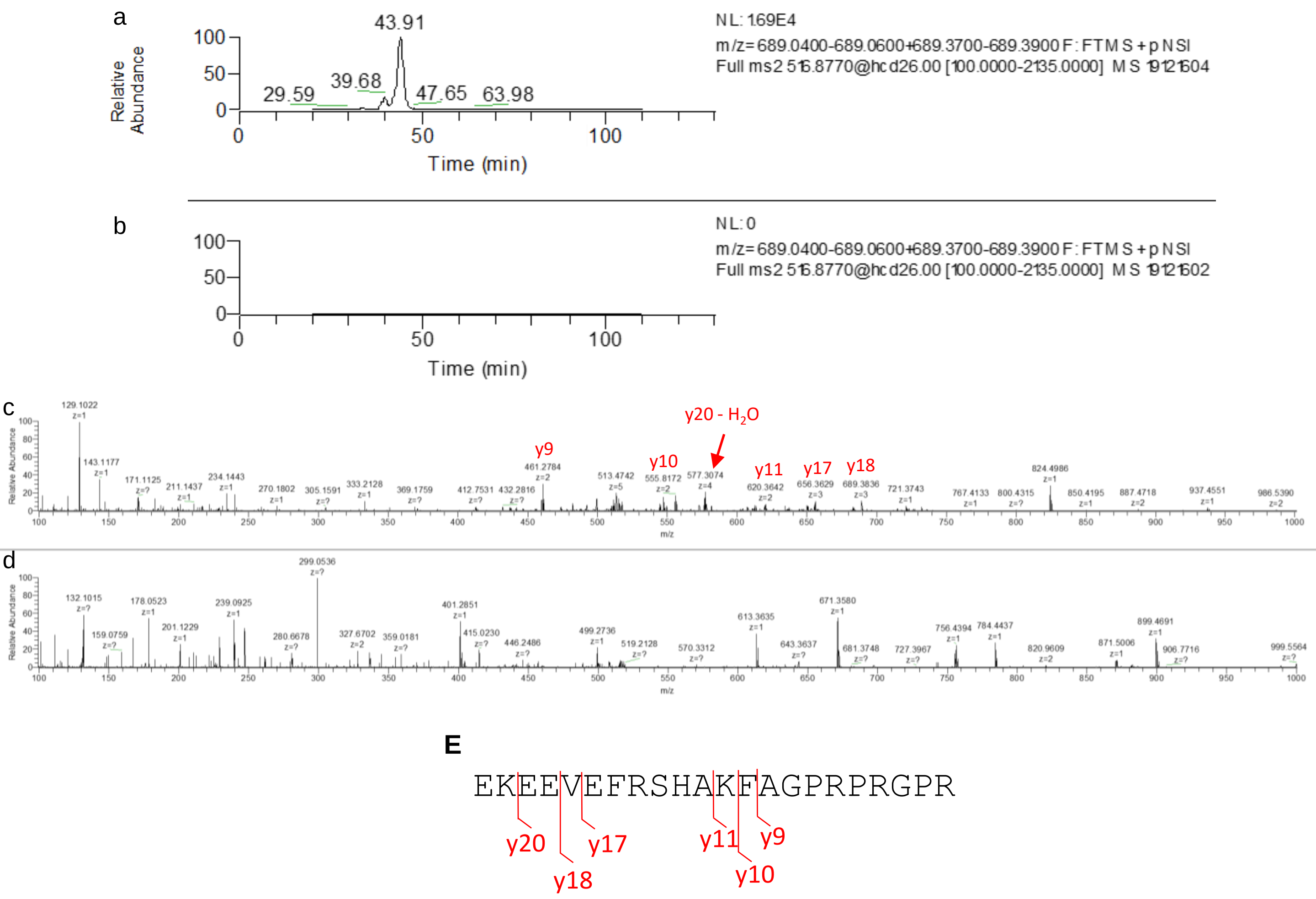

Supplementary figure 4: Searching for the N-terminal propeptide of proGip (Gip 22-43) in mouse islets.

a,b. Extracted ion chromatograms from a product ion scan monitoring for fragments of an ion with a *m/z* of 516.8770 in either an extract of homogenised mouse duodenum (a) or mouse islets (b). This 516.8770 precursor ion corresponds to the $[M+5H]^{5+}$ charge state of Gip 22-43.

c,d. Product ion spectra monitoring for fragments Gip 22-43 in homogenised mouse duodenum (c) and lysed mouse islets (d). These spectra display ions detected at retention times between 42.7-46.9 min for (a) and (b) respectively.

e. Sequence of mouse Gip 22-43 with y ions identified in (c) annotated. Gip 22-43 was detectable in mouse duodenum, as multiple y ions produced by the fragmentation of Gip 22-43 were detected at a retention time of 43.91 min. No b or y ions produced by the fragmentation of Gip 22-43 were detected at the same retention time in mouse islets.

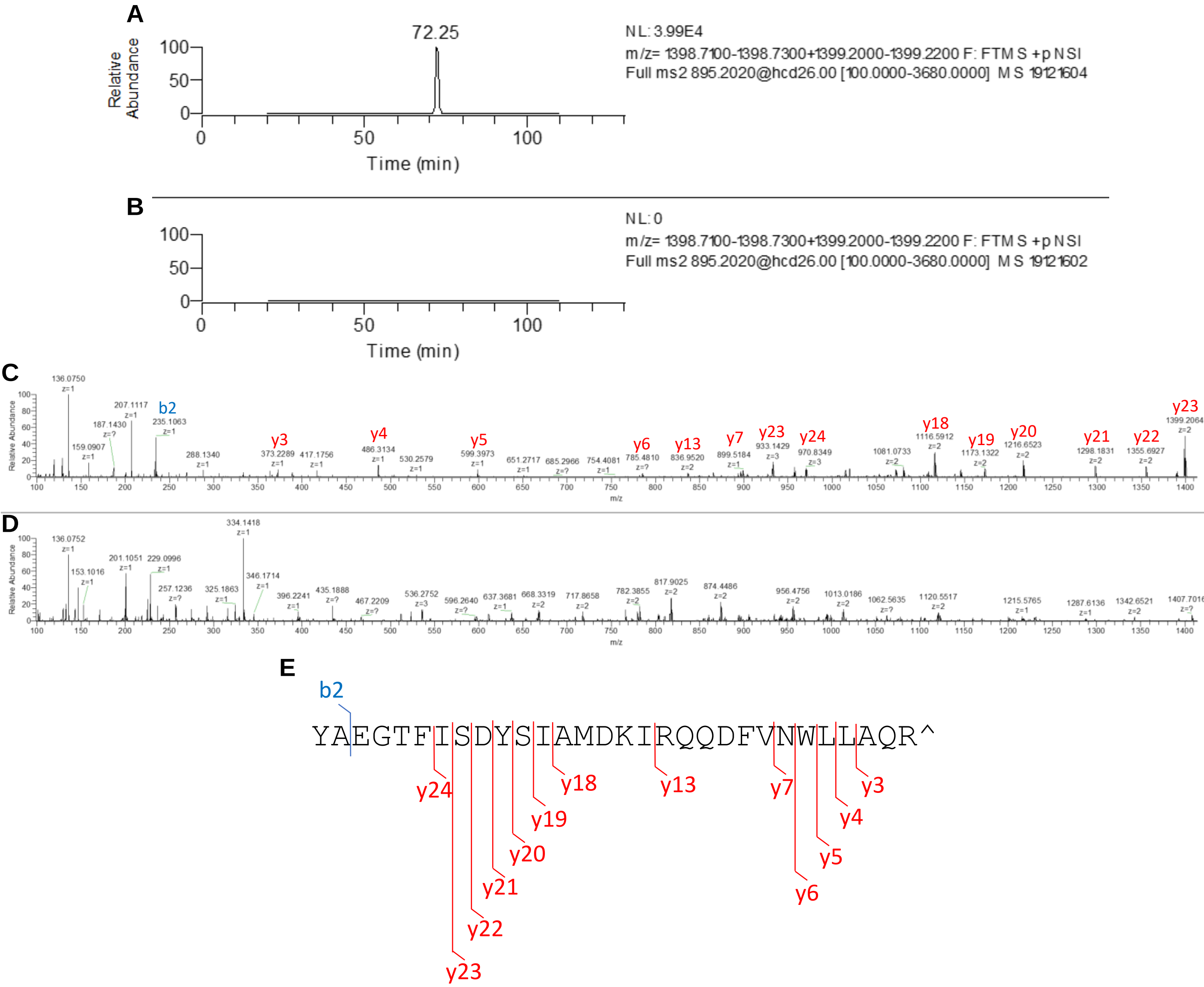

Supplementary figure 5: Searching for GIP (1-30) in mouse islets.

a,b. Extracted ion chromatograms from a product ion scan monitoring for fragments of an ion with a *m/z* of 895.2020 in either extracts of homogenised mouse duodenum (a) or mouse islets (b). This 895.2020 precursor ion corresponds to the $[M+4H]^{4}+$ charge state of GIP (1-30).

c,d. Product ion spectrums monitoring for fragments of GIP (1-30) in homogenised mouse duodenum (c) and lysed mouse islets (d). These spectra display ions detected at retention times between 70.9-74.4 min for (a) and (b) respectively.

e. Sequence of mouse GIP (1-30) with b and y ions identified in (c) annotated. ^ represents a C-terminal amidation. GIP (1-30) was detectable in mouse duodenum as multiple b and y ions from GIP (1-30) were detected at a retention time of 72.25 min. No b or y ions originating from GIP (1-30) were detected at the same retention time in mouse islets.

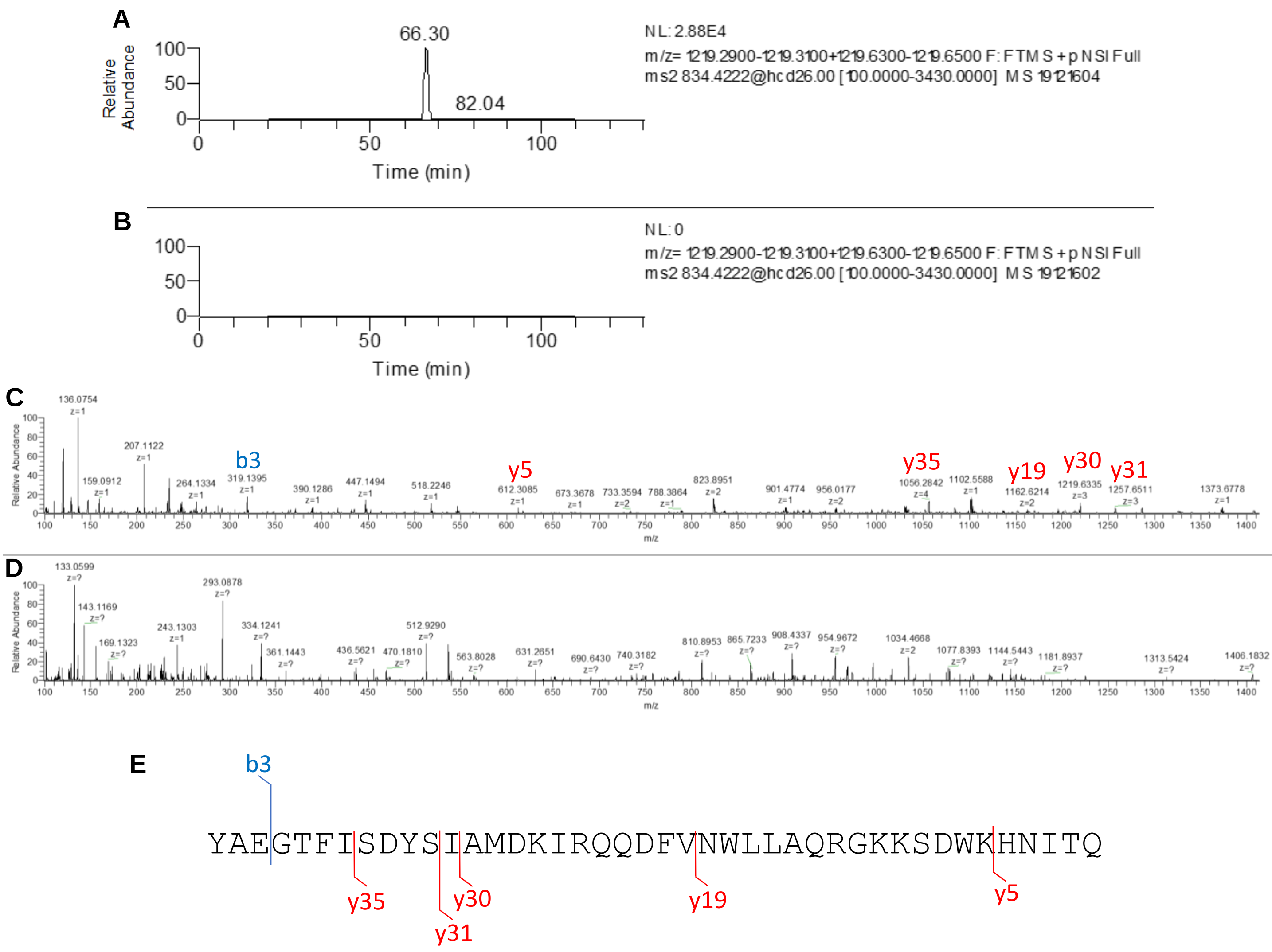

Supplementary figure 6: Searching for GIP (1-42) in mouse islets. (**A**, **B**) Extracted ion chromatograms from a product ion scan monitoring for fragments of an ion with a *m/z* of 834.4222 in either extracts of homogenised mouse duodenum (**A**) or mouse islets (**B**). This 834.4222 precursor ion corresponds to the $[M+6H]^{6+}$ charge state of GIP (1-42). Product ion spectrums monitoring for fragments of GIP (1-42) in homogenised mouse duodenum (**C**) and lysed mouse islets (**D**)**.** These spectra display ions detected at retention times between 62.9-68.5 min for **A** and **B** respectively. (**E**) Sequence of mouse GIP (1-42) with b and y ions identified in **C** annotated. GIP (1-42) was detectable in mouse duodenum as multiple b and y ions produced by the fragmentation of GIP (1-42) were detected at a retention time of 66.30 min. No b or y ions produced by the fragmentation of GIP (1-42) were detected at the same retention time in mouse islets.

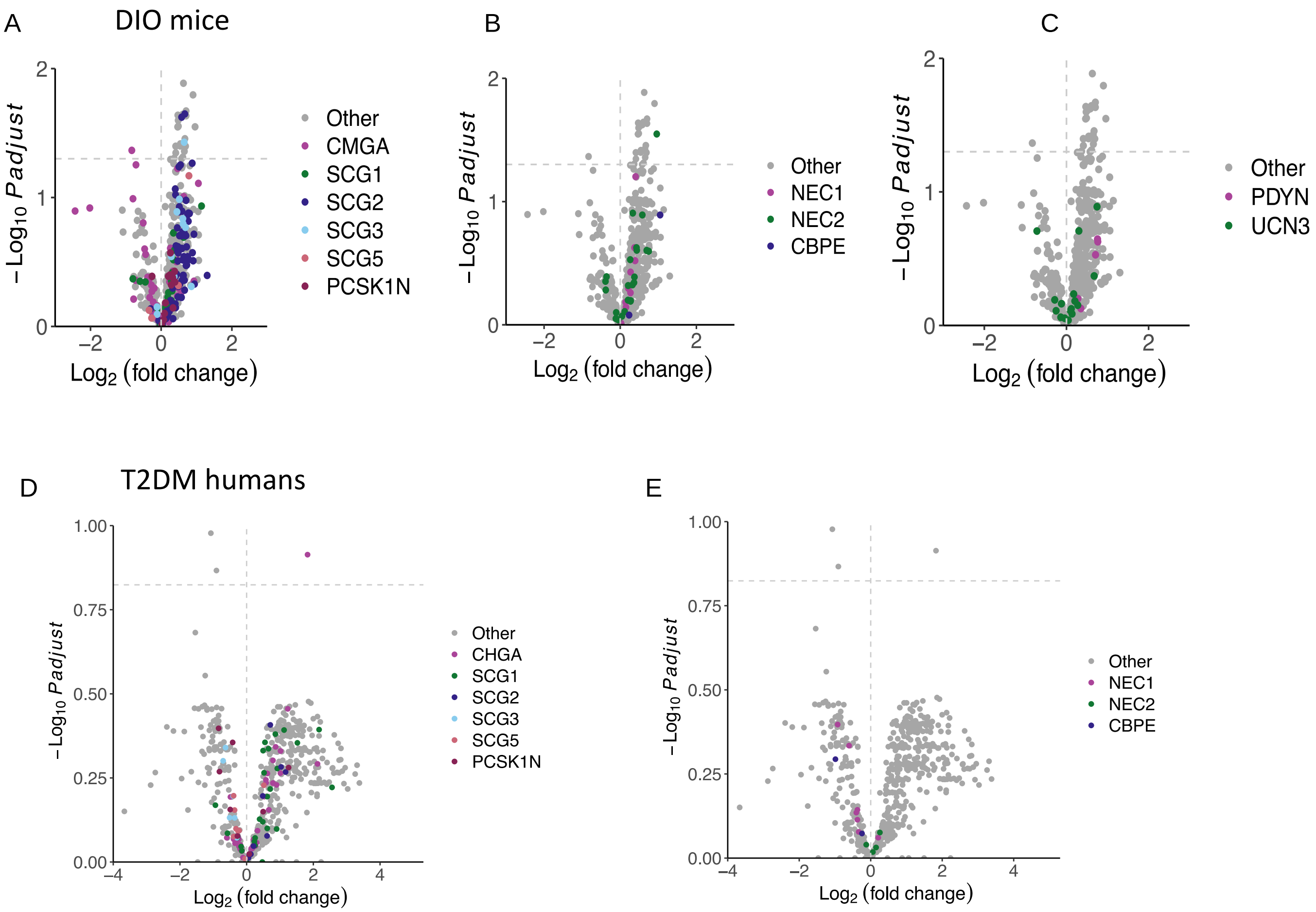

Suppl Figure 7: Peptidomic comparison of islets from DIO mice and T2D.

a-c. DIO Mice (continued from figure 4). Volcano plots displaying $\log_2$ fold change vs $-\log_{10}$ of the adjusted p value for each peptide. A positive log2 fold change indicates an increase in DIO mice. 715 t-tests were performed to analyse for significant differences in peptides matched between the groups with a permeation-based method used to adjust for multiple comparisons. Different peptides are coloured in each plot. Granin derived peptides in (a). Processing enzyme derived peptides in (b). PDYN and UCN3 derived peptides in (c).

d,e. T2D compared with non-diabetic controls (continued from figure 5). Volcano plot displaying $\log_2$ fold change vs $-\log_{10}$ of the adjusted p value for each peptide. A positive log2 fold change indicates an increase in T2D. Granin derived peptides coloured in (d) and processing enzyme derived peptides coloured in (e).
